# Oleic Acid-Based Self Micro-Emulsifying Delivery System for Enhancing Antifungal Activities of Clotrimazole

**DOI:** 10.3390/pharmaceutics14030478

**Published:** 2022-02-22

**Authors:** Ting-Lun Yang, Chien-Ming Hsieh, Ling-Jei Meng, Tsuimin Tsai, Chin-Tin Chen

**Affiliations:** 1 Department of Biochemical Science and Technology, College of Life Science, National Taiwan University, Taipei 106, Taiwan; f05b22054@ntu.edu.tw (T.-L.Y.); r08b22021@ntu.edu.tw (L.-J.M.); 2 School of Pharmacy, College of Pharmacy, Taipei Medical University, Taipei 110, Taiwan; cmhsieh@tmu.edu.tw; 3 FormuRx Pharmaceuticals Co., Ltd., Taipei 10617, Taiwan; tmtsai00@gmail.com

**Keywords:** *Candida albicans*, *Candida tropicalis*, clotrimazole, self micro-emulsifying drug delivery system, biofilm

## Abstract

Due to the increasing rate of drug resistance in *Candida* spp., higher doses of antifungal agents are being used resulting in toxicity. Drug delivery systems have been shown to provide an effective approach to enhance the efficacy and reduce the toxicity of antifungal agents. Oleic acid was revealed to effectively inhibit biofilm formation, hence reducing the virulence of *Candida albicans*. In this study, oleic acid-based self micro-emulsifying delivery systems (OA-SMEDDS) were developed for delivering clotrimazole (CLT). Based on the pseudo-ternary phase diagram and loading capacity test, the optimal ratio of OA-SMEDDS with CLT was selected. CLT-loaded OA-SMEDDS not only bears a higher drug loading capacity but also maintains good storage stability. The minimum inhibitory concentration (MIC_50_) of CLT-loaded OA-SMEDDS (0.01 μg/mL) in *Candida albicans* was significantly lower than that of CLT dissolved in DMSO (0.04 μg/mL). Moreover, we showed CLT-loaded OA-SMEDDS could effectively prevent biofilm formation and destroy the intact biofilm structure of *Candida albicans.* Furthermore, a CLT-loaded OA-SMEDDS gel was developed and evaluated for its antifungal properties. Disk diffusion assay indicated that both CLT-loaded OA-SMEDDS and CLT-loaded OA-SMEDDS gels were more effective than commercially available products in inhibiting the wild-type and drug-resistant species of *Candida*.

## 1. Introduction

*Candida albicans* represents a common symbiotic microorganism in humans. If the body’s immune system becomes suppressed, they can transform into pathogens and cause opportunistic infections in the host. In recent years, fungal infections in patients with immunodeficiency disorders have become an escalating problem. Irrespective of whether medical advances extend their life expectancy, the rising proportion of the elderly population also increases the risk of infection with opportunistic pathogens. Moreover, *C. albicans* infection is also common in patients treated with antibiotics, which alters the balance of flora in the body, resulting in the overgrowth of fungi and causing candidiasis [1]. The most common candidiasis caused by antibiotic treatment is vulvovaginal candidiasis (VVC) [2], which is mainly attributed to *C. albicans* (>85%) [3]. VVC impacts most females at least once in their lifespan (−70%), and 40–50% of them encounter a relapse [4,5]. The preponderance of evidence shows that *C. albicans* isolated from patients with uncomplicated or recurrent VVC (RVVC) is susceptible to most first-line antifungal agents, however, these drugs were reported to be clinically ineffective in eradicating Candida or treating RVVC [6]. Symptomatic vulvovaginal candidiasis can also be caused by non-*Candida albicans* (NAC) species, such as *Candida tropicalis*, *Candida glabrata, Candida parapsilosis* etc. [7]. Although the common species of NAC is *Candida glabrata* [8], some evidence indicates that recurrence rates of *Candida tropicalis* in candida vulvovaginitis measured twice that of *Candida albicans* [9], suggesting alternative dosage forms are needed for treating fungal infection.

Fluconazole is used as the first-line agent for VCC or RVCC; however, the increasing incidence of fluconazole-resistant *Candida* spp. in recent years has caused a treatment dilemma [10]. Clotrimazole (CLT) was reported to be comparable and superior to fluconazole in clinical studies for treating VCC [11,12]. CLT is usually administered vaginally as a cream, gel or suppository before bedtime for the treatment of VCC. However, common drawbacks of commercial CLT products include irritation, leakage of the formulation, and short residence time in the vaginal cavity [13]. In addition, there is inter- or intra-subject variation in vaginal absorption due to the poor solubility of CLT. CLT loaded in liposomal gel [14], cyclodextrin [15], nanostructured lipid carrier [16], microemulsion [17] and microemulsion-based gel [18] were developed to enhance the solubility and local activity in vivo. However, it is not clear whether these CLT formulations could affect the biofilm formation of *Candida albicans* and fluconazole-resistant *Candida* spp.

Biofilm, attaching to the surface of an inanimate object or living body, is formed by microorganisms and their secreted extracellular polymeric substances. The biofilm structure physically limits the diffusion of antimicrobial agents, which contributes to persistent chronic infections and resistance to antimicrobial agents [19]. Recent evidence shows the possibility of using pharmaceutic excipients to increase the activity of antibiotics against microbial biofilms. For example, oleic acids (OA) were shown to reduce filamentation and biofilm formation of *Candida albicans* without affecting the growth of planktonic cells [20]. In addition, Tween 80 (TW80) bears the ability to down-regulate the expression of fatty acid synthase, which is required for *Candida* spp. growth in the absence of exogenous fatty acids, thereby inhibiting virulence [21]. Although polyethylene glycol 400 (PEG 400) has no direct effect on biofilm formation, PEGylated antimicrobial drugs shows potential for overcoming biofilm-associated drug resistance [22].

Self micro-emulsifying drug delivery system (SMEDDS) presents a promising lipid-based formulation to improve solubility and bioavailability of lipophilic/hydrophobic active pharmaceutical ingredients (APIs). This drug delivery system is an isotropic mixture of oil, surfactant and co-solvent phase. As the dilution rate of water gradually increases, SMEDDS first transforms to gel form spontaneously, and then assembles into oil in water (o/w) microemulsion sized droplets under appropriate agitation. The formation of o/w microemulsion droplets escalate the interfacial surface area of hydrophobic or lipophilic agents for drug absorption [23]. In addition, o/w micro-emulsion droplets protect inner encapsulated compounds from the surrounding environment. Consequently, the chance of interaction between the APIs and substances in the aqueous phase is reduced, leading to the improvement of drug stability [24]. Due to additional kinetic and dynamic forces during the manufacturing process, the crystal form of internal excipients and APIs can be transformed into a metastable form. Hence, the amount of APIs that can be carried in SMEDDS far exceeds the saturated concentration [25], which provides the concentration driving force to accelerate the absorption ability of the APIs.

In this study, clotrimazole (CLT)-loaded OA-SMEDDS, which is composed of oleic acid (OA), Tween 80 (TW80), and polyethylene glycol 400 (PEG 400) was developed and further transformed into a gel form to investigate the antifungal activity. The optimal formulation of CLT-loaded OA-SMEDDS was selected based on a ternary phase diagram. The physiochemical properties of the optimal CLT-loaded OA-SMEDDS, including droplet size, size distribution (PDI) and stability, were further evaluated. The antifungal activity of CTL-loaded OA-SMEDSS was assessed in planktonic and biofilm cells of *Candida albicans*. The releasing profile of CTL-loaded OA-SMEDSS and OA-SMEDSS gel were evaluated by Franz cell diffusion test. Finally, the inhibitory zone in wild- and resistant- types of different *Candida* spp. was studied by disk diffusion assay.

## 2. Materials and Methods

### 2.1. Materials

Monobasic potassium phosphate (KH_2_PO_4_) was purchased from FUJIFILM Wako Pure Chemical Corporation, Osaka, Japan. Yeast peptone dextrose (TPD) and granulated agar were obtained from Difco, Detroit, MI, USA. Phosphate buffer saline (PBS) and Roswell Park Memorial Institute 1640 (RPMI 1640) medium were purchased from Sigma-Aldrich, St. Louis, MO, USA. Clotrimazole (>98%) was purchased from Tokyo Chemical Industry, Tokyo, Japan. Oleic acid was obtained from Emperor Chemical Co., Ltd., Taipei, Taiwan. Polysorbate 80 (Tween 80) was purchased from Kao Corporation, Wakayama, Japan. Polyethylene glycol 400 (PEG 400) was acquired from Scharlau Chemie, Barcelona, Spain. Clotrimazole commercial cream, Mycosten^®^, was purchased from Sinphar Pharmaceutical Co., Ltd., Yilan, Taiwan. Supor^®^ 200 PES Membrane Disc Filter was purchased from Pall Corporation, Portsmouth, UK.

### 2.2. Candida Strains and Growth Condition

*Candida albicans* SC5314 (ATCC MYA-2876D) and *Candida tropicalis* (ATCC MYA-3404) were generously gifted from Dr. Ching-Hsuan Lin, Department of Biochemical Science and Technology, National Taiwan University, Taipei, Taiwan. Fluconazole-resistant clinical *C. albicans* (2008 no. 22) and fluconazole-resistant clinical *C. tropicalis* (F2011ac003) were kindly provided by Dr. Yee-Chun Chen, Center of Infection Control, National Taiwan University Hospital, Taipei, Taiwan. These *Candida* cells were incubated in a YPD medium under aerobic conditions at 37 °C for 16–18 h. After centrifugation at 12,000 rpm for 2 min, the cell suspensions were harvested and washed at least 3 times with phosphate buffered saline, then adjusted to 10^7^ CFU/mL cells with RPMI 1640 medium for the following studies.

### 2.3. Clotrimazole Solubility Test

The solubility of CLT was evaluated in OA, TW80, and PEG 400 by the shake flask method. Briefly, an excess amount of the CLT was weighted into Eppendorf tubes and independently vortexed for approximately 1 min to create a muddy appearance. Slurries were mixed for 24 h at ambient temperature in aphotic conditions. Samples were then centrifuged at 3000 rpm for 15 min. The total supernatant was passed through a 0.22 µm pore size non-sterile filter, and then diluted with methanol. The methanol solutions were analyzed by UV-Visible Spectrophotometer DU8000 (Beckman Coulter, Brea, CA, USA) and detected at a wavelength of 272 nm. The calibration curve was elaborated by serially diluting the clotrimazole stock solution in methanol. All experiments were repeated three times.

### 2.4. Construction of Ternary Phase Diagram and Preparation of CLT-Loaded OA-SMEDDS and OA-SMEDDS Gel

A ternary phase diagram was constructed using SigmaPlot^®^ software version 12 (Systat Software, Inc., San Jose, CA, USA), consisting of OA (as oil phase), TW80 (as surfactant phase), and PEG 400 (as co-solvent phase) in the volume ratios of 10–70%, 20–80% and 5–55%, respectively. Briefly, each different volume fraction of excipients was mixed at 65 ± 0.5 °C and 1000 rpm for 30 min. The excipient mixture was added with an appropriate amount of CLT to another bottle, and then dispersed to a final concentration of 100 mg/mL under the above conditions. Among them, only pre-formulations with a transparent and clear appearance without any precipitation were selected for follow-up research.

CLT-loaded (OA-)SMEDDS gel was constructed from CLT-loaded OA-SMEDDS. Briefly, ddH_2_O was gradually added into the selected CLT-loaded OA-SMEDDS at a ratio of 5% until the ratio of SMEDDS to water reached 65:35 (*w*/*w*).

### 2.5. Micromeritic Properties and Stability Test of CLT-Loaded OA-SMEDDS

Optimized formulations of CLT-loaded OA-SMEDDS were diluted in 3000 dilution fold in Simulated Intestinal Fluid (SIFsp) with a pH of 6.8, which is described in the United States Pharmacopeia-National Formulary (USP26-NF21) [26] and RPMI Media 1640 for mimicking the dilution process in the lumen of gastrointestinal tract. The formed micro-emulsions were detected by an SZ-100 particle size analyzer (Horiba, Japan) to further investigate the particle size. Chemical stability of the drug loaded in OA-SMEDDS was assessed by quantifying the total amount of CLT in OA-SMEDDS during storage at 25 °C and 40 °C for at least 2 months.

### 2.6. Viscosity Test of CLT-Loaded OA-SMEDDS Gel

The viscosity of CLT-loaded OA-SMEDDS gel was detected by a VISCO-895 viscometer (Atago Co., Inc., Tokyo, Japan) using an A1 spindle at ambient temperature and 10 rpm. The maximum torque was set at 70%.

### 2.7. Antifungal Susceptibility Test

The susceptibility test for MIC_50_ was determined by the Clinical and Laboratory Standards Institute (CLSI) broth microdilution M27-A3 method [27]. Briefly, *Candida albicans* were diluted in RPMI 1640 medium containing 2% glucose and 0.165 mol/L 3-(*N*-morpholino) propanesulfonic acid with pH of 7.0 until the concentration was around 10^4^ CFU/mL. Different concentrations (0, 0.01, 0.02, 0.04, 0.08, 0.16 μg/mL) of CLT in DMSO and CLT-loaded OA-SMEDDS were then added into the culture of diluted *C. albicans* for further incubation at 37 °C. After 48 h of incubation, the MIC_50_ value was defined by the lowest concentration at which the cultural medium remains clear when read visually in microtiter plates.

### 2.8. Effects of CLT-Loaded OA-SMEDDS on Biofilm

The biofilms of *C. albicans* were prepared using a silicone model [28]. Briefly, sterilized silicon membranes (PR72034-06N, Woodland, CA, USA) were placed into a 12-well plastic plate and incubated with fetal bovine serum at 37 °C for 18 h. To investigate the inhibitory effect on the biofilm formation, 10^7^ CFU/mL of *C. albicans* in RPMI 1640 medium was added into the plates containing different concentrations (0, 0.01, 0.1, 1, 10 μg/mL) of CLT-loaded OA-SMEDDS and further incubated at 37 °C for 48 h under 100 rpm shaking. The silicone membranes were washed with PBS and dried for 48 h, then weighed to calculate the biomass of each biofilm sample.

To investigate the destructive effect of the matured biofilm, 10^7^ CFU/mL of *C. albicans* was added into the plates containing silicon membranes and incubated with fetal bovine serum at 37 °C for 90 min under 100 rpm shaking. The silicone membranes were washed with PBS and further incubated with RPMI 1640 for another 48 h at 37 °C under 100 rpm shaking. The matured biofilms developed on the silicone membranes were further incubated with different doses (0, 1, 10, 30, 50 μg/mL) of CLT-loaded OA-SMEDDS at 37 °C for 48 h. The silicon membranes were washed with PBS and dried for 48 h, then weighed to determine the biomass of each biofilm sample.

### 2.9. Disc Diffusion Assay

The process of disc diffusion assay was performed according to CLSI guidelines (document M44-2) [27]. Briefly, *C. albicans,* fluconazole-resistant *C. albicans, C. tropicalis,* fluconazole-resistant *C. tropicalis* were cultured in a 37 °C incubator with shaking at 250 rpm until reaching the logarithmic phase. Then, a sterilized cotton swab was used to moisten *Candida* spp. suspension and the suspension was smeared evenly on the RPMI 1640 agar medium. Sterile filter paper was placed on the culture medium and 0.1 mg or 0.5 mg of experimental drugs was added. Following this, the medicated medium was placed in a 37 °C incubator for 48 h, and the size of the inhibition zone was recorded.

### 2.10. Franz Cell Diffusion Test

The releasing profile of CLT in OA-SMEDDS and OA-SMEDDS gel was performed by Franz cell diffusion test. PES membrane was selected due to the hydrophilic properties of SMEDDS and SMEDDS gel. Due to the hydrophobicity of APIs, at least 50% ethanol was added into the buffer of the donor compartment to accelerate diffusion efficiency [29,30]. Meanwhile, the pH value of the buffer medium was 5.8 to mimic the acidic condition on the skin, caused by secretions from sudoriferous glands, sebum, and the breakdown of fatty acids by *Staphylococcus* in the epidermis [31]. Briefly, SMEDDS or SMEDDS gel was spread evenly on the PES membrane, which is clamped between the receptor and donor cell. A volume of 50% ethanol in PBS (pH 5.8) was filled in the receptor cell at 32 ± 0.5 °C and stirred at 500 rpm. A 1 mL sample was removed at different time intervals, while the isothermal and equal volume medium was supplemented. Each sample was qualified by UV spectrophotometer.

## 3. Results

**Figure 1:**
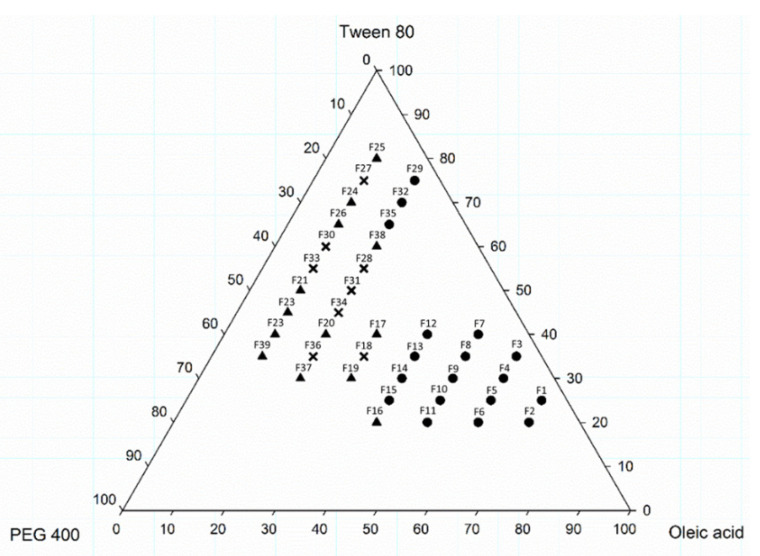
Ternary phase diagram of CLT-loaded OA-SMEDDS containing oleic acid (OA), Tween 80 (TW80), and polyethylene 400 (PEG 400). ●: Transparent appearance. ▲: Precipitation occurred. **×**: Both precipitation and a turbid appearance were exhibited. All samples were visually observed after 15 days of storage at ambient temperature.

To determine the appropriate ratio of OA, TW80 and PEG400 for preparing CLT-loaded OA-SMEDDS, we constructed a ternary phase diagram in the presence of CLT. As shown in Figure 1, when the volume fraction of OA is 10–70% (*v*/*v*), TW80 belongs to 20–80% (*v*/*v*), and PEG 400 exists in 5–55%, a clear, uniform, and transparent of CLT-loaded OA-SMEDDS without precipitation could be obtained. However, after 15 days of storage at ambient temperature, drug precipitation was identified in the samples of F16–F28; F30; F31; F33; F34; F36; F37. Therefore, these pre-formulation groups were not considered for further studies. In addition, application of OA of up to 25% concentration may cause irritation of guinea pig skin [32]. In this regard, formulas F1–F19 were excluded as well. As high doses of PEG 400 may cause nephrotoxicity, the content of PEG 400 should not exceed 30% [33]. Therefore, only three pre-formulations (F29, F32 and F35) were selected to perform the following studies.

**Figure 2:**
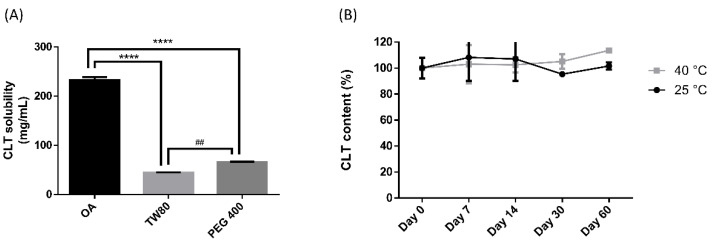
Solubility and drug degradation of CLT. (**A**) CLT solubility in oleic acid (OA), Tween 80 (TW80), and polyethylene glycol 400 (PEG 400). (**B**) Two-month chemical degradation curve of CLT in F35 at 25 and 40 °C. Each value represents the mean ± standard deviation from three independent experiments. **** *p* < 0.0001 as compared to CLT solubilized in OA. ## *p* < 0.01 as compared to CLT solubilized in TW80.

Next, in order to study the maximum capacity of CLT in OA-SMEDDS, we examined the solubility of CLT in OA, TW80, and PEG 400, respectively. As shown in Figure 2A, compared to TW80 (44.15 mg/mL) and PEG 400 (65.71 mg/mL), CLT exhibits the highest solubility in OA (231.41 mg/mL). These results indicate that the theoretical solubility of CLT in F29, F32 and F35 is about 82–85 mg/mL. As the loading dose of CLT is 100 mg/mL, we further examined the SMEDDS stability of the F29, F32 and F35 in supersaturated conditions. Only F35 remained stable without precipitation after 30 days of storage, indicating that F35 OA-SMEDDS bears a better stability than other formulas. Furthermore, the contents of CLT in F35 remained at the same level after 60 days of storage at 25 °C and 40 °C (Figure 2B). These results indicate the chemical stability of CLT could be maintained for at least 2 months in F35 formula. Table 1 shows the micrometric properties of F35 formula in Simulated Intestinal Fluid (SIFsp) and RPMI 1640 medium. The particle size of F35 formula was 12.1 nm and 84 nm in SIFsp and RPMI 1640, respectively, indicating it could self-emulsify to numerous nano-size droplets in the dissolution media. These results demonstrate that F35 can not only improve the dispersibility of CLT in vitro, but also theoretically enhance drug absorption rates in organisms.

We then further examined the in vitro antifungal activity of CLT-loaded F35 formula against wild-type *C. albicans* SC5314. As shown in Table 2, the MIC_50_ of CLT dissolved in DMSO measured 0.04 μg/mL. In contrast, the MIC_50_ of CLT-loaded F35 OA-SMEDDS significantly dropped to 0.01 μg/mL, approximately four-fold lower. It was shown that lipid substances might bear microbicidal activity due to its disruption of the cell membrane [34]. However, DMSO and OA-SMEDDS itself did not affect the growth of *C. albicans*, suggesting CLT formulated in OA-SMEDDS can increase its antifungal activity.

**Figure 3:**
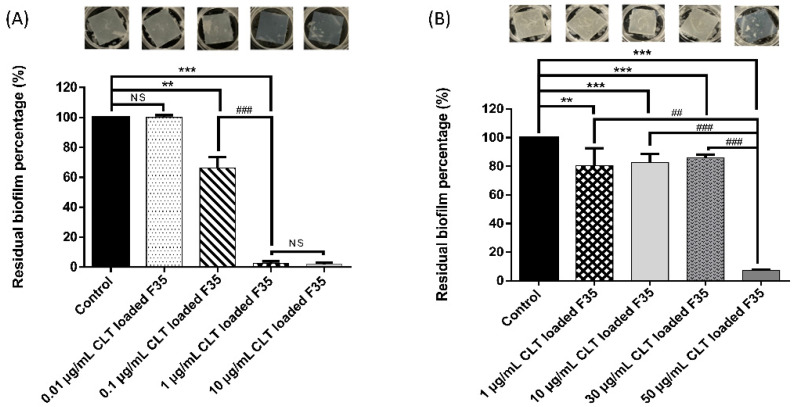
The effect of F35 with different CLT loading doses against *C. albicans* biofilm. (**A**) The result of *C. albicans* biofilm formation inhibitory tests by different loading doses of CLT in F35. (**B**) The degree of CLT-F35 causing *C. albicans* biofilm destruction. Each value represents the mean ± standard deviation from three independent experiments. ** *p* < 0.01 *** *p* < 0.001 as compared to F35 without drug. ## *p* < 0.01 ### *p* < 0.001 as compared to F35 with 1 μg/mL CLT loading dose.

Owing to its dense and protected environment as well as its different phenotypic properties, *C. albicans* biofilm is resistant to most antifungal agents. Therefore, we further examined whether CLT-loaded F35 OA-SMEDDS could exert an inhibitory effect on *C. albicans* biofilm which was formed on the silicone model [28]. As shown in Figure 3A, the ability of *C. albicans* biofilm formation was significantly suppressed when the CLT loading dose in F35 OA-SMEDDS increased to 0.1 μg/mL. No biofilm formation could be identified as the CLT loading dose was elevated to 1 μg/mL. Despite the fact that CLT-loaded F35 can inhibit biofilm formation, we further investigated whether CLT-loaded F35 can eradicate the fully developed biofilm structure. As shown in Figure 3B, 20% of the preformed biofilm was significantly eradicated in the presence of 1–30 μg/mL CLT and the eradication ratio further soared to 70% when the CLT loading dose in F35 was elevated to 50 μg/mL. These results clearly demonstrate that CLT formulated in F35 OA-SMEDDS can not only prevent biofilm formation but also eradicate the existing biofilm of *C. albicans*.

The antifungal activity of CLT-loaded F35 was further evaluated by disc diffusion assay using *C. albicans*, *C. tropicalis,* and drug-resistant strains. For comparison, the F35 formulation without CLT and a commercial cream, Mycosten^®^, were used. As shown in Table 3, the diameter of the inhibition zone formed by F35 containing 0.1 mg CLT was around 37.3 mm, 21.0 mm, 23.0 mm, and 21.0 mm in *C. albicans*, fluconazole-resistant *C. albicans*, *C. tropicalis,* and fluconazole-resistant *C. tropicalis,* respectively. Furthermore, the zone of inhibition increased with increasing CLT concentration. Moreover, the inhibition zone of commercial Mycosten^®^ was significantly smaller than that of CLT-loaded F35 under the same drug concentrations against wild-type and drug-resistant strains (Figures S1 and S2 of Supplementary Materials). No inhibition zone was observed in F35 without CLT, suggesting no inhibitory effect of F35 formula alone.

Due to the high fluidity of SMEDDS, adherence to the target tissue for topical application is difficult to achieve. It was shown that a series of optically isotropic and viscous gel phases may appear before being converted into spherical oil droplets upon dilution of a SMEDDS formula with an aqueous medium [35]. In this study, we found F35 formula could be converted into gel form after the addition of 35% (*w*/*w*) deionized water. As shown in Table 4, the viscosity of CLT-loaded F35 gel is about 23 times greater than that of CLT-loaded F35, suggesting molecular rearrangement might occur during the addition of aqueous phase.

**Figure 4:**
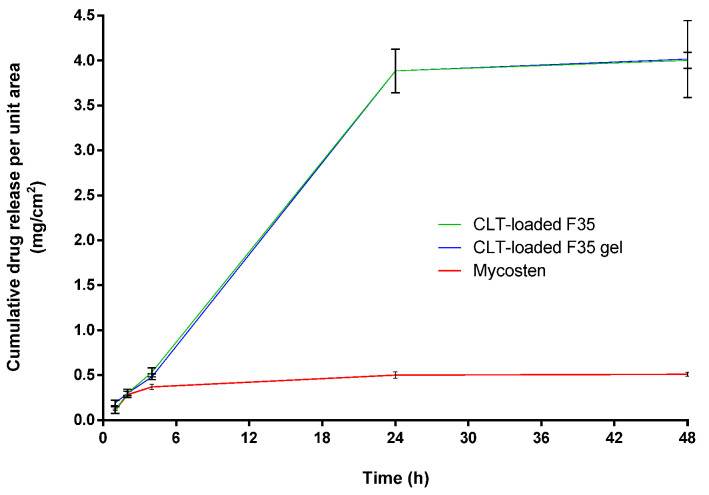
The releasing profile of CLT-loaded F35, CLT-loaded F35 gel, and CLT commercial product, Mycosten^®^ in Franz diffusion cells for 48 h. Each value represents the mean ± standard deviation from three independent experiments.

In the drug release study (as shown in Figure 4), the releasing profile of CLT in CLT-loaded F35 and CLT-loaded F35 gel reached the maximum cumulative drug concentration after 24 h. Moreover, the CLT amount released from both CLT-loaded F35 and CLT-loaded F35 gel is about 40 times greater than that of the commercially available cream, Mycosten^®^.

The antifungal properties of CLT-loaded F35 gel were further examined in a disk diffusion assay. As shown in Table 5 and Figure S3 of Supplementary Materials, the size of the inhibition zone formed by F35 gel containing 0.1 mg CLT measured about 37 mm, 30.5 mm, 32 mm, and 27 mm in *C. albicans*, fluconazole-resistant *C. albicans*, *C. tropicalis,* and fluconazole-resistant *C. tropicalis,* respectively. While the inhibition zone of Mycosten^®^ was significantly smaller (13 to 19.5 mm) than those of CLT-loaded F35 gel against *C. albicans*, *C. tropicalis,* and drug-resistant strains.

## 4. Discussion

CLT has been used as an antifungal agent for *C. albicans* infection. In addition, the clinical cure rate of CLT in the treatment of VCC was reported to be similar to fluconazole [36]. However, the solubility and toxicity impede the further development of this drug. SMEDDS was shown as a promising platform to enhance solubility of hydrophobic or lipophilic compounds, and to facilitate absorption by organisms. The properties of SMEDDS relate to the composition and ratio of the excipients, that influence stability and efficiency of the formulation and APIs. In this study, a series of OA-based SMEDDS formulas loaded with CLT were established (Figure 1). One of these CLT-loaded OA-SMEDDS, F35, exhibited a small particle size in the dispersibility test and maintained a clear and precipitate-free appearance for over 60 days (Figure 2). Moreover, there was no CLT degradation in F35 observed after storage at 40 °C for 60 days, suggesting the possibility of using F35 formula to preserve the chemical stability of CLT. This CLT-loaded F35 SMEDDS exerts a significant antifungal activity against *C. albicans* as shown by the quantitative method of susceptibility testing (Table 2) as well as biofilm studies (Figure 3). Finally, using disc diffusion assay, we demonstrated that F35 formula exerts a significant antifungal activity against wild-type and drug-resistant *Candida* spp. (Table 3 and Table 4).

It was demonstrated that the particle size of SMEDDS may be affected by the substances in the medium during the dissolution study [37]. In the dissolution medium, we discovered F35 could further self micro-emulsify into larger nano-level droplets in the RPMI medium compared to the SIFsp medium (Table 1). RPMI 1640 contains glucose, pH indicator, salts, amino acids, and vitamins, while the components of SIFsp are KH_2_PO_4_ and NaOH. Ricardo F. et al. reported that the covalent coupling of amino acid side chains can provide emulsifying characteristics [38]. Therefore, we speculate that the difference in particle size of the microemulsion might relate to amino acids present in the RPMI 1640 medium. Bruna et al. reported that nanoparticles smaller than 100 nm can easily penetrate through the cell wall into the microbial cell [39,40]. After self micro-emulsification, the particle size of F35 measured 84 nm and 12 nm in RPMI and SIFsp dissolution media, respectively. In this regard, the CLT-loaded F35 nanoparticles in the dissolution medium could enter into the microbial organisms and exert pharmacological effects. Moreover, according to Fick’s first law [41], the donor side of drug concentration will significantly increase due to the improved solubility following self micro-emulsification. Consequently, the accumulation of the drug at the apical side increases. Since SMEDDS belongs to a supersaturated delivery system, they can generate a higher concentration gradient next to microbial organisms, thereby enhancing the driving force of passive diffusion and increasing the absorption ability of the drugs.

As shown in Table 3, the inhibition zone of CLT-loaded F35 was larger than that of the commercial product, Mycosten^®^, indicating CLT-loaded F35 as a promising pre-formulation for further development. However, the fluidity of F35 OA-SMEDDS might limit its feasibility as a topical medication, such as for treatment of vulvovaginal candidiasis. Liquid crystals (LC), so called mesophases, represent a state of matter that blends the structures and properties of conventional liquids and solid crystals. It was shown that the structures of lyotropic LCs contribute to the stabilization of the diluted lipid-based formulation and enhance drug releasing profile [42]. Hosmer and Esposito et al. demonstrated its potential for topical application due to the viscous enhancement [43,44]. In this regard, purified water-based SMEDDS liquid crystal (SMEDDS-LC) gel can be employed to improve the API retention for mucosal treatment. It was revealed that the lyotropic liquid crystal could be formed with the addition of 10–40% (*w*/*w*) of water into SMEDDS [45]. Moreover, published literature also reported the water content in the liquid crystal should be less than 35% (*w*/*w*) to prevent the influence of drug releasing profile [46]. To enhance the viscosity of CLT-loaded F35, we added 35% (*w*/*w*) of water into CLT-loaded F35 to generate a lyotropic liquid crystal-like gel form. As shown in Table 4, the viscosity of SMEDDS indeed can be significantly increased by the addition of the aqueous phase, suggesting the retention time of SMEDDS-gel can increase at the topical site. Furthermore, in the Franz cell diffusion assay, there was no difference in the CLT releasing profile between CLT-loaded F35 and CLT-loaded F35 gel, suggesting the drug release from these two formulations would be theoretically similar in clinical use. In fact, as shown in Table 3 and Table 5, the antifungal activity of CLT-loaded F35 gel in wild- and drug-resistant types of *C. albicans* and *C. tropicalis* are similar to CLT-loaded F35, implying that the gel form did not affect the antifungal ability.

## 5. Conclusions

In this study, CLT-loaded OA-SMEDDS was developed and exhibited an antifungal ability against planktonic and biofilm cells of *C. albicans*. Moreover, the CLT-loaded OA-SMEDDS was further converted into gel form with significant antifungal activity against wild-type and drug-resistant *C. albicans* and *C. tropicalis*. These results demonstrate the potential of CLT-loaded SMEDDS pre-formulation use for treating local infections such as VVC or RVVC.

## Figures and Tables

**Table 1:** Micrometric properties of CLT-loaded F35 after self-emulsifying in RPMI 1640 and SIFsp medium.

	RPMI 1640	SIFsp
Particle size (nm)	84 ± 24.2	12.1 ± 5.3
PDI value	0.233	0.342
Size range	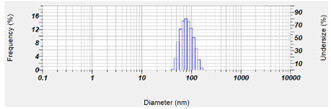	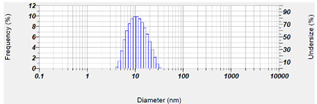

Each value represents the mean ± standard deviation from three independent experiments.

**Table 2:** MIC_50_ of *C. albicans* with CLT in different formula.

Strain	Antimicrobial Agent	Inoculum	Growth Medium	MIC_50_ (μg/mL)
*C. albicans*	CLT-loaded F35	5×10^3^	RPMI 1640	0.01
	CLT in DMSO	5×10^3^	RPMI 1640	0.04
	DMSO only	5×10^3^	RPMI 1640	Not observed
	F35 without drug	5×10^3^	RPMI 1640	Not observed

Each value represents the average of three independent experiments.

**Table 3:** Inhibition zone of wild-type and drug-resistant *C. albicans* as well as *C. tropicalis* treated with CLT-loaded F35 and commercial cream, Mycosten^®^.

Strain	Formula	Drug Conc./Disk (mg)	Inhibition Zone (mm)
*C. albicans*	CLT-loaded F35	0.5	45.0 ± 2.0
		0.1	37.3 ± 3.0
	Mycosten^®^	0.5	25.0 ± 0.1
		0.1	19.5 ± 3.5
	F35 without drug	0	0
Fluconazole-resistant *C. albicans*	CLT-loaded F35	0.5	34.5 ± 1.5
		0.1	21.0 ± 6.0
	Mycosten^®^	0.5	0
		0.1	0
	F35 without drug	0	0
*C. tropicalis*	CLT-loaded F35	0.5	33.0 ± 1.0
		0.1	23.0 ± 4.0
	Mycosten^®^	0.5	15.0 ± 1.0
		0.1	10.5 ± 1.5
	F35 without drug	0	0
Fluconazole-resistant *C. tropicalis*	CLT-loaded F35	0.5	27.5 ± 2.5
		0.1	21.0 ± 4.0
	Mycosten^®^	0.5	12.0 ± 1.0
		0.1	10.5 ± 2.5
	F35 without drug	0	0

Each value represents the average of three independent experiments.

**Table 4:** Viscosity and representative appearance of CLT-loaded F35 and CLT-loaded F35 gel at ambient temperature.

	CLT-Loaded F35	CLT-Loaded F35 Gel
Viscosity	279.3 ± 0.9 mPass	6422.6 ± 10.7 mPass
Representative Appearance	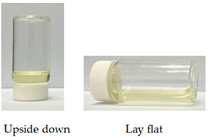	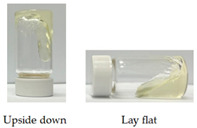

Each value represents the average of three independent experiments.

**Table 5:** Inhibition zone of wild-type and drug-resistant *C. albicans* as well as *C. tropicalis* with CLT-loaded F35 gel and commercial cream, Mycosten^®^.

Strain	Formula	Drug Conc./Disk (mg)	Inhibition Zone (mm)
*C. albicans*	CLT-loaded F35 gel	0.1	37.0 ± 3.0
	Mycosten^®^	0.1	19.5 ± 3.5
	F35 gel without drug	0	0
Fluconazole-resistant *C. albicans*	CLT-loaded F35 gel	0.1	30.5 ± 2.5
	Mycosten^®^	0.1	0
	F35 gel without drug	0	0
*C. tropicalis*	CLT-loaded F35 gel	0.1	32.0 ± 1.0
	Mycosten^®^	0.1	13.5 ± 1.5
	F35 gel without drug	0	0
Fluconazole-resistant *C. tropicalis*	CLT-loaded F35 gel	0.1	27.0 ± 6.0
	Mycosten^®^	0.1	13.0 ± 2.0
	F35 gel without drug	0	0

Each value represents the average of three independent experiments.

## Supplementary Materials

The following are available online at https://www.mdpi.com/article/10.3390/pharmaceutics14030478/s1, Figure S1: Representative plate of disk diffusion assay showing inhibition zones against wild-type and fluconazole resistant *C. albicans* by F35 loading with or without CLT and commercial CLT cream, Mycosten^®^. Figure S2: Representative plate of disk diffusion assay showing inhibition zones against wild-type and fluconazole resistant *C. tropicalis* by F35 loading with or without CLT and commercial CLT cream, Mycosten^®^. Figure S3: Representative plate of disk diffusion assay showing inhibition zones against wild-type and fluconazole resistant *C. albicans* and *C. tropicalis* by F35 gel loading with or without 0.1 mg CLT and commercial CLT cream, Mycosten^®^.
