# Supplementary material for: Oleic Acid-Based Self Micro-Emulsifying Delivery System for Enhancing Antifungal Activities of Clotrimazole
Source: Pharmaceutics. 2022 Feb 22;14(3):478. doi: 10.3390/pharmaceutics14030478 (PMC8951188; doi:10.3390/pharmaceutics14030478)
Supplement: Supplementary file 1 [file pharmaceutics-14-00478-s001.zip › pharmaceutics-1592472-supplementary.pdf]

pharmaceutics

MDPI

Supplementary Materials

Oleic Acid-Based Self Micro-Emulsifying Delivery System for Enhancing Antifungal Activities of Clotrimazole

Ting-Lun Yang [1], Chien-Ming Hsieh [2], Ling-Jei Meng [1], Tsuimin Tsai [3] and Chin-Tin Chen [1,*]

[1] Department of Biochemical Science and Technology, College of Life Science, National Taiwan University, Taipei 106, Taiwan; f05b22054@ntu.edu.tw (T.-L.Y.); r08b22021@ntu.edu.tw (L.-J.M.)
[2] School of Pharmacy, College of Pharmacy, Taipei Medical University, Taipei 110, Taiwan; cmhsieh@tmu.edu.tw
[3] FormuRx Pharmaceuticals Co. Ltd., Taipei 10617, Taiwan; tmtsai00@gmail.com
* Correspondence: chintin@ntu.edu.tw; Tel.: +886-2-3366-9487; Fax: +886-2-3366-2271

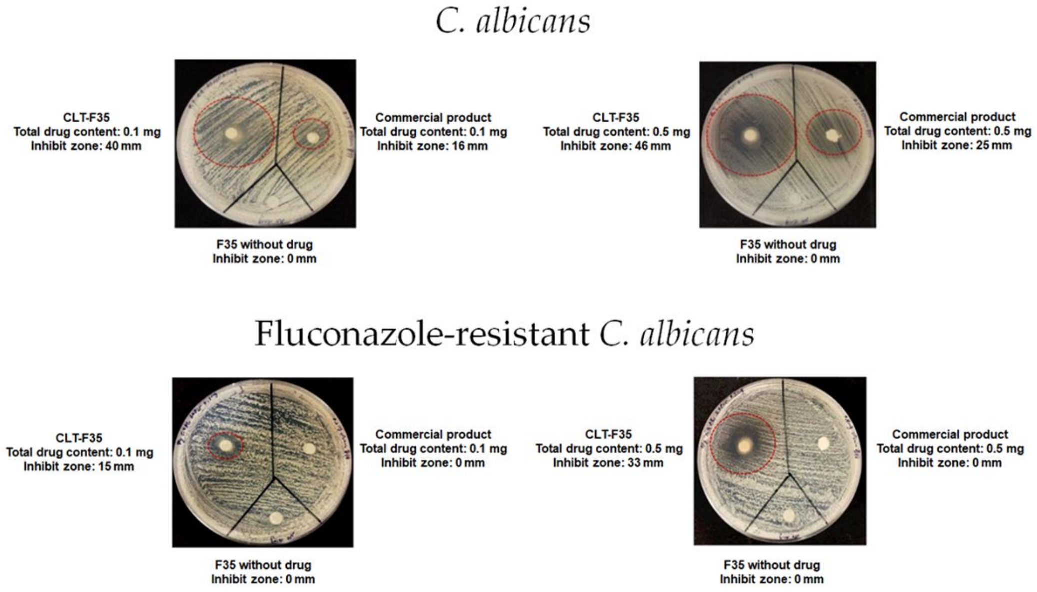

Figure S1. Representative plate of disk diffusion assay showing inhibition zones against wild-type and fluconazole resistant *C. albicans* by F35 loading with or without CLT and commercial CLT cream, Mycosten®. After inoculating 2×10^6 CFU/mL of wild-type and fluconazole-resistant *C. albicans* on RPMI 1640 medium, each group was treated with F35 and Mycosten® containing 0.1 mg or 0.5 mg CLT, then incubate at 37 °C for 48 h.

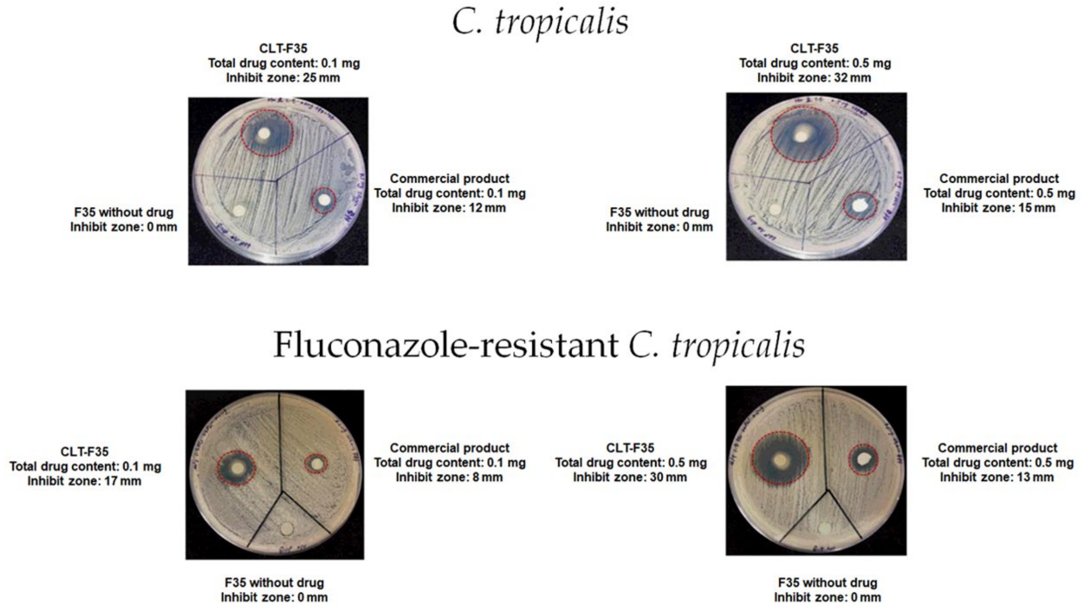

Figure S2. Representative plate of disk diffusion assay showing inhibition zones against wild-type and fluconazole resistant *C. tropicalis* by F35 loading with or without CLT and commercial CLT cream, Mycosten®. After inoculating 2 × 10^6 CFU/mL of wild-type and fluconazole-resistant *C. tropicalis* on RPMI 1640 medium, each group was treated with F35 and Mycosten® containing 0.1 mg or 0.5 mg CLT, then incubate at 37°C for 48 h.

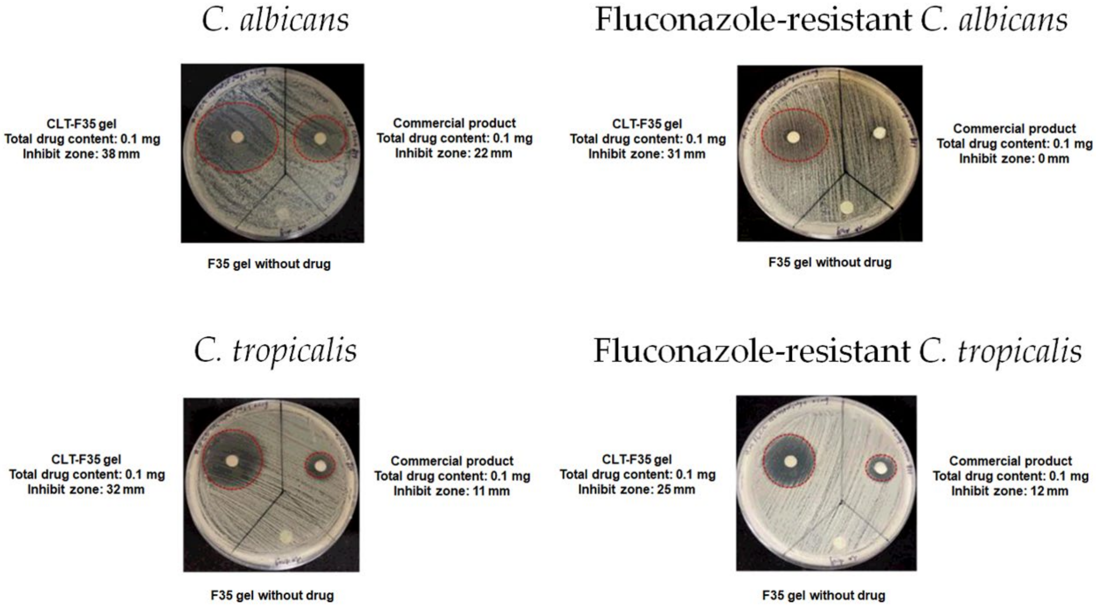

Figure S3. Representative plate of disk diffusion assay showing inhibition zones against wild-type and fluconazole resistant *C. albicans* and *C. tropicalis* by F35 gel loading with or without 0.1 mg CLT and commercial CLT cream, Mycosten®. After inoculating 2 × 10^6 CFU/mL of *C. albicans*, fluconazole-resistant *C. albicans*, *C. tropicalis* and fluconazole-resistant *C. tropicalis* on RPMI 1640 medium, each group was treated with F35 gel and Mycosten® containing 0.1 mg CLT, then incubate at 37 °C for 48 h.
